# One-and-a-half year old child with leukocoria

**DOI:** 10.4103/0974-620X.71908

**Published:** 2010

**Authors:** Kishore Hanumanthappa

**Affiliations:** Department of Ophthalmology, Al-Nahda Hospital, Muscat, Oman

A one-and-a-half year old Omani girl was noted by her parents to have a white reflex in her right eye [Figure 1]. There was no sign of intraocular inflammation [Figure 2]. Pre-natal history was insignificant. The child was otherwise healthy. Family history was unremarkable.

**Figure 1:**
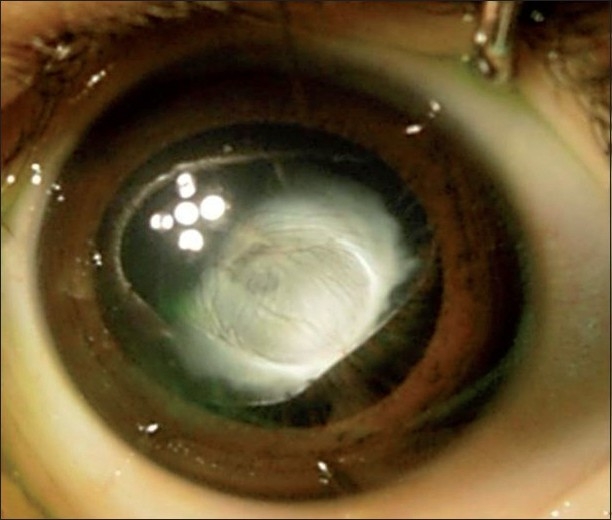
Anterior segment photograph OD

**Figure 2:**
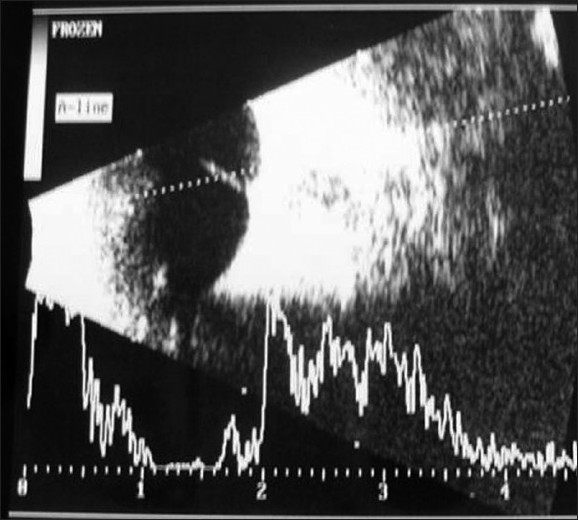
B-scan ultrasound OD

## Questions:

1. Describe the findings in Figure 1.
2. Describe the findings in Figure 2.
3. What is the most likely diagnosis?

## Answers:

1. Retrolental plaque with posterior subcapsular lens cataract, with vasularization.
2. Microphthalmic eye with small amplitude echoes from retina extending anteriorly.
3. Persistent hyperplastic primary vitreous.

## Comments

Persistent hyperplastic primary vitreous (PHPV) is a term used to describe a wide spectrum of congenital anomalies. They range from anterior PHPV (cataract with persistent fetal vasculature) to posterior PHPV (condensed vitreous running from optic disk to ora serrata).

Anterior PHPV, which is seen in the patient presented, may be associated with a microphthalmic eye with shallow anterior chamber, retrolenticular fibrovascular plaque, elongated ciliary process and occasional intralenticular hemorrhage, and elevated intraocular pressure (IOP). When the retrolenticular plaque is vascular, it may cause intralenticular hemorrhage, and bleeds into vitreous if cut surgically. In some patients, the lens may be clear initially, but with time becomes cataractous. In some patients, the lens can undergo spontaneous absorption while in others a spontaneous break in the posterior lens capsule can cause lens to become intumescent causing elevation in IOP. Retinal involvement can occur secondary to contraction of retrolental plaque resulting in traction on vitreous base and peripheral retina.

